# Genomic Characterization of a Carbapenemase-Producing, Extensively Drug-Resistant Klebsiella michiganensis Strain from a Renal Abscess Patient

**DOI:** 10.1128/mra.00825-22

**Published:** 2022-11-02

**Authors:** Li-Miao Hu, Xiao-Tuan Zhang, Xi Zeng, Yajun Chen, Logen Liu, Guo-Qing Li

**Affiliations:** a Department of Gastroenterology, The Second Affiliated Hospital, Hengyang Medical School, University of South China, Hengyang, Hunan, China; b Clinical Laboratory, The Second Affiliated Hospital, Hengyang Medical School, University of South China, Hengyang, Hunan, China; c Clinical Research Center, The Second Affiliated Hospital, Hengyang Medical School, University of South China, Hengyang, Hunan, China; d Key Laboratory of Molecular Diagnosis and Precision Medicine in Hengyang, Hengyang, Hunan, China; Loyola University Chicago

## Abstract

We describe an extensively drug-resistant Klebsiella michiganensis strain, Kmfe267, which was originally isolated from a renal abscess patient. The strain carries the blaNDM-5 gene, which encodes a New Delhi metallo-β-lactamase. The complete genome of the strain contains a 5.9-Mb chromosome and 5 plasmids.

## ANNOUNCEMENT

Klebsiella pneumoniae is an opportunistic pathogen that causes infection in immunocompromised patients. However, as a carbapenemase-producing and extensively drug-resistant species, it has become a major challenge (1, 2). Furthermore, the spread of Klebsiella pneumoniae carbapenemase (KPC) or New Delhi metallo-β-lactamase (NDM) to other species of the Klebsiella genus has been reported and shows distinct features of infection (3–5), with strains often being mistaken as K. pneumoniae in clinics. Here, we characterized a Klebsiella michiganensis strain that was isolated from a patient with a renal abscess. This strain had resistance to multiple antibiotics and carried NDM-5 carbapenemase. The wide spread of antibiotic resistance and possible invasiveness to K. michiganensis needs our urgent attention.

Urine and left kidney drainage samples were collected from a patient with a renal abscess. The samples were inoculated onto Columbia blood, MacConkey, and chocolate agar plates with a 10-μL inoculating loop and cultured at 37°C in a 5% CO_2_ incubator overnight. Species identification and antibiotic susceptibility testing of the strain were performed using the Vitek 2 Compact system (bioMérieux). Bacteria that grew on all three plates from both samples were identified as K. pneumoniae by the instrument. The strain possessed multidrug resistance, including carbapenems. The research was approved by the Medical Research Ethics Committee of the Second Affiliated Hospital, University of South China (approval number 2021K041602).

The isolated strain was cultured in LB broth at 37°C in a shaking incubator, and genomic DNA was extracted with a GeneJET kit (Thermo Fisher Scientific). DNA (400 ng) was barcoded using a rapid barcoding sequencing kit (SQK-RBK004; Oxford Nanopore Technologies) and then sequenced with the MinION system with a SpotON flow cell (R9.4.1; Oxford Nanopore Technologies). Base calling, quality filtering, and barcode trimming were performed with Guppy v3.4.4 (https://community.nanoporetech.com) (parameter settings: –c dna_r9.4.1_450bps_fast.cfg –qsocre_filtering –trim_barcodes –barcodes_kits SQK-RBK004). A total of 1,052,962 reads were generated, and the *N*_50_ value is 12.8 kb. Genomic DNA was also subjected to Illumina library preparation with the NEBNext Ultra DNA library preparation kit, and sequencing was performed on an Illumina HiSeq 2000 system with a 2 × 150-bp flow cell (Sangon, China), which generated 2.8 million reads. The raw data were quality filtered with Trimmomatic v0.39 (6). The sequencing depth with both sequencing methods was about 120×. Unicycler v0.4.8 (7) was used for *de novo* hybrid assembly of the circularized chromosome and plasmid sequences. Default parameters were used for all software tools except as mentioned. The genome consists of a 5.9-Mb chromosome and five plasmids (Table 1). The GC content of the whole genome is 55.5%. Species identification with Kleborate software v2.0.0 (8) identified the strain as K. michiganensis. Genome annotation was performed with the NCBI Prokaryotic Genome Annotation Pipeline (PGAP) v5.3 (9).

**TABLE 1:** Information on the assembled genome

Genomic element	GenBank accession no.	Size (bp)	GC content (%)	Resistance feature
Chromosome	CP071393.1	5,924,548	56.1	
Plasmid pKmfe267-1	CP071394.1	251,953	47.3	Two resistance islands
Plasmid pKmfe267-2	CP071395.1	170,909	50.1	
Plasmid pKmfe267-3	CP071396.1	46,161	46.7	*bla*_NDM–5_
Plasmid pKmfe267-4	CP071397.1	2,772	40.4	
Plasmid pKmfe267-5	CP071398.1	1,943	48.7	

**FIG 1:**
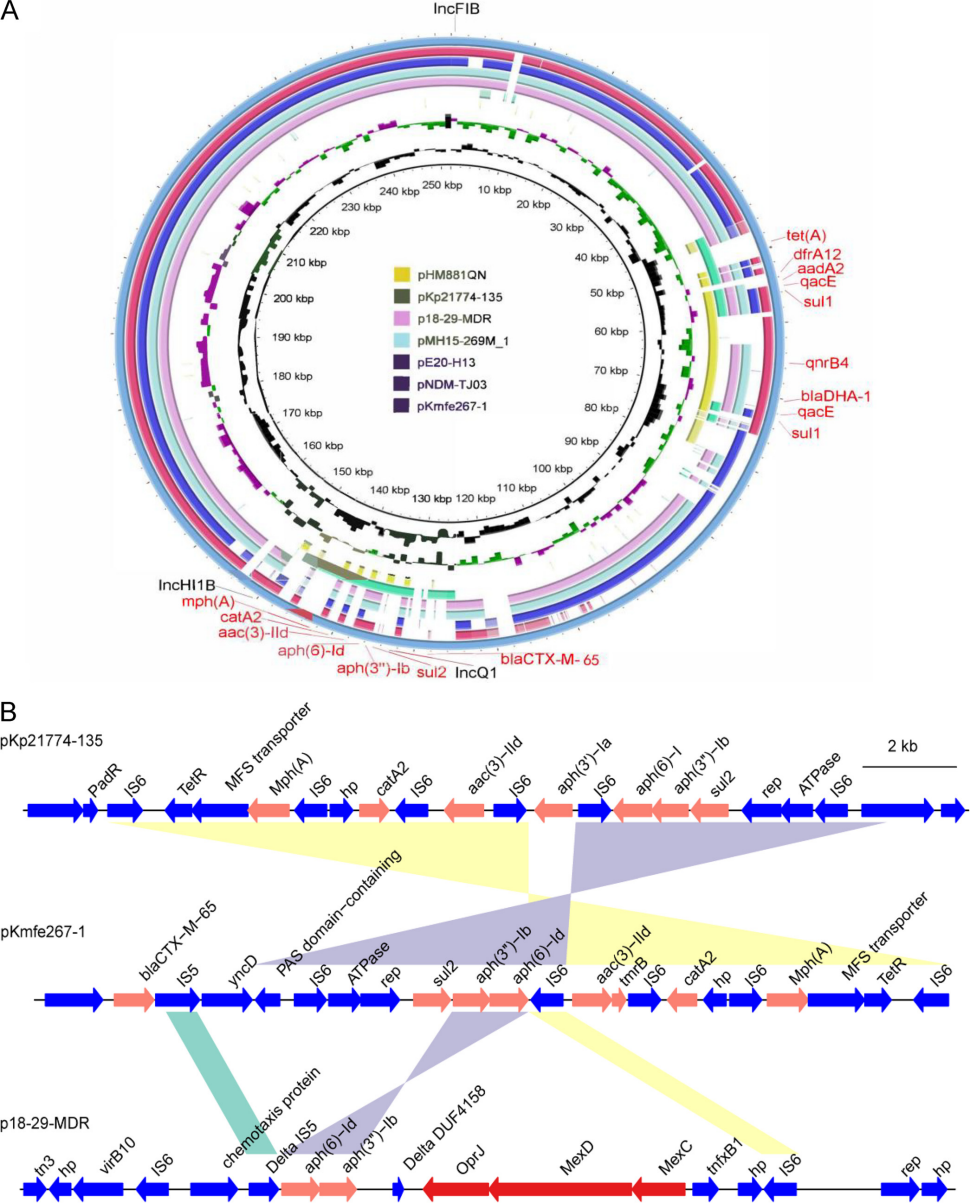
Identification of two resistance islands in the plasmid pKmfe267-1 and genomic comparison of the second resistance island. (A) Plasmid map and characteristics. The two gene islands conferred resistance to most antibiotics except carbapenem, whose resistance was conferred by another plasmid carrying approximately 46-kb sequences. The plasmids showed that the genes most similar to the first resistance islands were pHM881QN (GenBank accession number NZ_LC055503) and pNDM-TJ03 (GenBank accession number NZ_MG845201), while the plasmid most similar to the second resistance islands was pKp21774-135 (GenBank accession number NZ_MG878868). However, p18-29-MDR (GenBank accession number NZ_MK262712) and pMH15-269M_1 (GenBank accession number NZ_AP023338), two plasmids containing tigecycline resistance from the RND efflux pump gene cluster, share the plasmid backbone with pKmfe267-1. The image was created with BRIG software v0.95 (11). (B) Genomic comparison of the second resistance island of pKmfe267-1 with pKp21774-135 and p18-29-MDR. The data showed that pKp21774-135 and pKmfe267-1 shared the resistance island (upper and middle segments). Resistance genes are colored orange, and shadings indicate alignments of gene homology. p18-29-MDR, which shared the plasmid backbone with pKmfe267-1, may acquire the RND efflux pump gene cluster (MexD, MexC, or OprJ in the bottom gene segment, highlighted in red). The image was created with genoPlotR package v0.8.11 (12).

The resistance genes of this strain were identified with ResFinder v4.0 (10), and the pKmfe267-1 plasmid consists of two antibiotic resistance islands (Fig. 1A). A BLAST search suggested that many tigecycline resistance plasmids (taking p18-29-MDR, which shares the plasmid backbone with pKmfe267-1, as an example) (Fig. 1A) seemed to acquire a gene cluster of the resistance-nodulation-division (RND) efflux pump in the second resistance island (Fig. 1B).

### Data availability.

The raw sequencing data for the strain were uploaded to the Sequence Read Archive (SRA), and the accession numbers for Illumina and Nanopore sequencing data are SRR20999548 and SRR20999547, respectively. The assembled genome was also uploaded to the NCBI assembly database with accession number GCA_017348855, with the chromosome and plasmids all included.
